# LUCAT1-Mediated Competing Endogenous RNA (ceRNA) Network in Triple-Negative Breast Cancer

**DOI:** 10.3390/cells13221918

**Published:** 2024-11-19

**Authors:** Deepak Verma, Sumit Siddharth, Ashutosh S. Yende, Qitong Wu, Dipali Sharma

**Affiliations:** Department of Oncology, Johns Hopkins University School of Medicine and the Sidney Kimmel Comprehensive Cancer Center at Johns Hopkins, Baltimore, MD 21287, USA

**Keywords:** LUCAT1, competing endogenous RNA network, triple-negative breast cancer, microRNAs, long noncoding RNAs

## Abstract

Breast cancer is a heterogeneous disease comprising multiple molecularly distinct subtypes with varied prevalence, prognostics, and treatment strategies. Among them, triple-negative breast cancer, though the least prevalent, is the most aggressive subtype, with limited therapeutic options. Recent emergence of competing endogenous RNA (ceRNA) networks has highlighted how long noncoding RNAs (lncRNAs), microRNAs (miRs), and mRNA orchestrate a complex interplay meticulously modulating mRNA functionality. Focusing on TNBC, this study aimed to construct a ceRNA network using differentially expressed lncRNAs, miRs, and mRNAs. We queried the differentially expressed lncRNAs (DElncRNAs) between TNBC and luminal samples and found 389 upregulated and 386 downregulated lncRNAs, including novel transcripts in TNBC. DElncRNAs were further evaluated for their clinical, functional, and mechanistic relevance to TNBCs using the lnc2cancer 3.0 database, which presented LUCAT1 (lung cancer-associated transcript 1) as a putative node. Next, the ceRNA network (lncRNA–miRNA–mRNA) of LUCAT1 was established. Several miRNA–mRNA connections of LUCAT1 implicated in regulating stemness (LUCAT1-miR-375-Yap1, LUCAT1-miR181-5p-Wnt, LUCAT1-miR-199a-5p-ZEB1), apoptosis (LUCAT1-miR-181c-5p-Bcl2), drug efflux (LUCAT1-miR-200c-ABCB1, LRP1, MRP5, MDR1), and sheddase activities (LUCAT1-miR-493-5p-ADAM10) were identified, indicating an intricate regulatory mechanism of LUCAT1 in TNBC. Indeed, LUCAT1 silencing led to mitigated cell growth, migration, and stem-like features in TNBC. This work sheds light on the LUCAT1 ceRNA network in TNBC and implies its involvement in TNBC growth and progression.

## 1. Introduction

With an estimated 310,720 new cases in 2024, breast cancer remains the most prevalent cancer among women in the United States [1]. Despite profound advancements in diagnostics, therapeutics, and molecular screening, the mortality associated with breast cancer remains high, with an estimated 42,250 breast cancer-related deaths in 2024 [1]. Breast cancer is inherently a heterogeneous disease with multiple distinct subtypes identified based on pathological, histological, and molecular features. The molecular heterogeneity is imparted by the expression levels of estrogen receptor (ER), progesterone receptor (PR), and human epidermal growth factor receptor 2 (HER2) amplification, along with varied prevalence of several mutations [2]. Luminal A subtype is hormone receptor-positive and HER2-negative with low expression levels of Ki67. Luminal B subtype tumors are hormone receptor-positive with either HER2-positive or -negative status with high expression of Ki67. HER2-enriched subtype tumors have higher expression of HER2 along with the presence or absence of hormone receptors. Triple-negative breast cancers (TNBCs) are characterized by the loss of ER, PR, and HER2 [2], which leaves no targeted therapeutic option. In terms of occurrence, luminal A is the most commonly identified breast cancer, followed by luminal B- and HER2-enriched subtypes, and while TNBCs represent only ~11% of all breast cancer cases [2], they are considered the most aggressive. In addition to the lack of targeted therapies and its inherently aggressive nature, TNBC is also marred by its molecular heterogeneity, with six subgroups—basal-like 1 (BL1), basal-like 2 (BL2), mesenchymal (M), mesenchymal stem-like (MSL), immunomodulatory (IM), and luminal androgen receptor (LAR) [3]—that contribute to higher mortality [4]. Efforts are ongoing to further understand the molecular differences among the TNBCs and other breast cancer subtypes to uncover tangible nodes that may be targeted. While earlier studies focused on gene expression alterations in TNBC, recent studies have been investigating the role of noncoding RNAs (ncRNAs) in cancer progression.

Indeed, in addition to the protein-coding genes, the noncoding repertoire plays a crucial role in cancer development. Noncoding RNAs (ncRNAs), earlier known as junk transcriptional products, are the functional regulatory molecules participating in complex biological networks with other RNAs and proteins [5]. Based on nucleotide (nt) length, ncRNAs can be divided into small noncoding RNAs (sncRNAs, 19–25 nt) and long noncoding RNAs (lncRNAs; ≥200 nt). sncRNAs mainly include microRNAs (miRNAs) that target the protein-coding mRNAs via the formation of the RISC complex, thus inhibiting mRNA translation and ensuing biological functions [6]. lncRNAs are also involved in various biological processes and disease development, such as epithelial–mesenchymal transition (EMT), metastasis, migration, proliferation, invasion, and tumorigenesis [7]. It is now well established that ncRNAs, including miRNAs, lncRNAs, and circular RNAs, regulate gene expression, and their aberrant expression results in the initiation and progression of various cancer types, including breast cancer [8,9,10,11,12,13]. Over the last decade, increased advancement in genomic technologies has led to the discovery of gene signatures involving ncRNAs pertaining to breast cancer molecular subtypes [14]. The involvement of ncRNAs with mRNAs has been frequently studied as a competing endogenous RNA network (ceRNA network) where lncRNAs may compete with miRNAs and inhibit the binding of miRNAs to their corresponding mRNA targets, thereby reversing miR-mediated suppression of mRNA levels leading to increased mRNA expression [15]. This approach, known as competing endogenous RNA networks, recruits a wide range of RNA species to target one or more RNA types and modulate the gene expression. In the case of tumor progression, lncRNA-mediated sponging of miRNAs leads to overexpression of oncogenes. This lncRNA–miRNA–mRNA (oncogene) mechanism has been noted in the progression and metastasis of various cancers, including colon, prostate, liver, and breast [8,15,16]. The ceRNA networks hold prognostic significance in lung adenocarcinoma, with TMPO-AS1-hsa-let-7c-5p-LDHA regulating aerobic glycolysis leading to enhanced tumor growth [17]. Interestingly, ceRNA networks influence proliferation, epithelial–mesenchymal transition, migration, invasion, autophagy, and apoptosis in urological, gynecological, gastrointestinal, and brain cancer [18]. The ceRNA networks also impact cancer stem cells [19]. Of note, ceRNA networks have emerged as post-translational regulators that impact multiple steps of cancer progression as well as response to therapy, metastatic progression, and recurrent disease.

In this study, we aimed to examine the ceRNA networks predominantly present in TNBCs in comparison to luminal subtypes in an effort to identify the lncRNA–miRNA–mRNA regulatory network(s) that may have functional importance in TNBCs. To this end, we conducted differential gene expression analysis of TNBCs and luminal tumors to identify the differentially expressed mRNAs and lncRNAs, followed by ceRNA network establishment. We observed that lncRNA LUCAT1 forms a ceRNA network in TNBC involving several miRs and their gene targets with oncogenic function. Inhibiting LUCAT1 leads to inhibition of growth, migration, and stem-like features in TNBC. Further, high LUCAT1 and low associated miR(s) as well as high LUCAT1 and high associated mRNA(s) expression levels associate with poor overall survival in TNBC patients, indicating that the LUCAT1 ceRNA network in TNBC may have mechanistic and functional significance warranting further investigation.

## 2. Material and Methods

### 2.1. Clinical Samples, RNA-Sequencing Data, and Identification of Differentially Expressed lncRNAs in TNBC

RNA-sequencing data were retrieved for 52 TNBC and 35 luminal breast cancer samples from the GSE192341 dataset. Read counts and clinical information were collected to analyze the samples. Further, sample reads were assembled in a data matrix to investigate differential gene expression through web-based integrated the Differential Expression and Pathway analysis (iDEP.95) shiny tool (Supplementary Table S1) [20]. iDEP utilizes DESeq2 to calculate DEGs. DEseq2 takes feature counts or read counts as input and uses a generalized linear model (gamma–Poisson distribution) to normalize the data before analyzing the logarithmic fold change; *p*-value is calculated by applying the Wald test [21]. Differentially expressed lncRNAs (DElncRNAs) were identified from the DEseq2 result file through the data mining ensemble biomart tool [22] utilizing user-defined parameters (±≥1.5 fold change, Supplementary Table S2).

### 2.2. Prediction of lncRNA for ceRNA Network and Construction of ceRNA Network

lnc2cancer3.0 [23] is a collection of experimentally supported lncRNAs and their mechanistic, functional, and clinical relevance in human cancers. We examined TNBC DElncRNA from the GSE192431 dataset within all three categories (functional, mechanistic, and clinical) using lnc2cancer, and the results are shown in a Venn diagram. LUCAT1 was the only TNBC DElncRNA that was present in all the categories (Supplementary Table S3). Further, expression of LUCAT1 was examined in publicly available TNBC samples through bc-GenExMiner v5.1 [24]. We constructed the lncRNA–miRNA–mRNA network through a user-defined approach using the lncACTDb [25] database. The potential interactions among lncRNAs, mRNA, and miRNA showed that a given lncRNA could target both miRNAs and mRNAs. We constructed a lncRNA–miRNA–mRNA network that could be clinically important for TNBC.

### 2.3. GSEA of LUCAT1-Correlated Genes and CCLE Data Analysis

Correlation analysis for LUCAT1 with all the DEGs in GSE192341 was performed using the “rcorr” function in R. The correlation cutoff was calculated by √2/n, and normalized reads of all the highly correlated genes (*n* = 714 r^2^ ≥ 0.30) (Supplementary Table S4) were used to perform GSEA [26] using hallmark in cancer (C1) and oncogene (C6) signatures (Supplementary Table S5). We performed DE analysis on mRNA and miRNA data of CCLE [27] breast cancer cell lines (Supplementary Table S6) by comparing the TNBC cell lines to all other subtypes of breast cancer using iDep0.95. We applied a focused approach and only queried the expression profiles of mRNA and miRNA that were associated with LUCAT1 in ceRNA network analysis.

### 2.4. Cell Culture and Reagents

The human triple-negative breast cancer cell lines MDA-MB-468, HCC1806, Hs578t, and HCC1937 [28] were procured from the American Type Culture Collection (ATCC, Manassas, VA, USA), revived from early-passage liquid nitrogen vapor stocks as required, and maintained at 37 °C in 5% CO_2_ and 95% humidity. All the cell lines were authenticated via short tandem repeat testing. No mycoplasma contamination was noted. For Western blot, anti-Bcl2-xL, anti-Bax, and anti-total-PARP were purchased from Cell Signaling Technology, Beverly, MA, USA. Mouse monoclonal β-actin was procured from Sigma-Aldrich, St. Louis, MO, USA. MTT (3-(4,5-dimethylthiazol-2-yl)-2,5-diphenyltetrazolium bromide) was procured from Sigma Aldrich, St. Louis, MO, USA. Chemiluminescent peroxidase substrate was procured from GE Healthcare, UK. The si-LUCAT and si-scrambled-control were procured from Thermo Fisher Scientific, Waltham, MA, USA.

### 2.5. Transfection, Cell Viability, Colony-Formation, and Trypan Blue Exclusion Assays

Cells were transfected with si-LUCAT and si-scramble-control using Lipofectamine 2000 (Thermo Fisher Scientific, Waltham, MA, USA) following the manufacturer’s instructions. MTT cell viability assays were performed to determine the percentage of metabolically active MDA-MB-468 cells in the presence and absence of LUCAT1 following the protocol mentioned earlier [29]. In brief, scramble-control and LUCAT1 siRNA-transfected MDA-MB-468 cells were seeded in 96-well plates at an initial density of 5 × 10^3^ cells/well for 48 h. After incubation, MTT reagent (3-(4,5-dimethylthiazol-2-yl)-2,5-diphenyltetrazolium bromide) (Sigma-Aldrich, St Louis, MO, USA) was added to each well at a final concentration of 0.5 mg/mL and incubated for additional 3 h at 37 °C for formazan crystal formation, which was then dissolved in DMSO (Sigma-Aldrich, St Louis, MO, USA), and the absorbance was measured at 570 nm using a plate reader. The data are presented as a percentage of control and means ± standard deviation. Colony-formation assays were performed following a protocol mentioned earlier [29,30]. Scramble-control and LUCAT1 siRNA-transfected TNBC cells were trypsinized and 500 cells/well were seeded in 12-well plates for 10 days. Cell culture medium replenishment was undertaken every third day. The colonies were fixed with formalin and stained with 0.1% crystal violet, air-dried, and images were captured using digital camera. For trypan blue dye exclusion assays, scramble-control and LUCAT1 siRNA-transfected MDA-MB-468 cells were trypsinized, resuspended in trypan blue dye, and counted using an automated hemocytometer (Countess 3, Thermo Fisher Scientific, Waltham, MA, USA) according to the protocol mentioned earlier [31]. Live cells that did not take up the dye were counted (scramble control = 35,500/200 µL; LUCAT1SiRNA = 14,500/200 µL). The total number of live cells is presented in bar graphs.

### 2.6. Transwell-Migration Assay

Transwell-migration assay was performed to evaluate the migration potential of scramble-control and LUCAT1 siRNA-transfected TNBC cells following the protocol mentioned earlier [32]. In brief, scramble-control and LUCAT1 siRNA-transfected TNBC cells were seeded in the top chambers of the transwell with an 8 µm filter containing serum-free medium. The lower chambers of the transwell were filled with 10% serum media and the cells were allowed to migrate through the transwell filters for 48 h. Non-migrated cells were removed using cotton swabs and the migrated cells were fixed and stained with 0.05% crystal violet. Migrated cells were imaged using a microscope and the images were captured.

### 2.7. Semiquantitative and Quantitative RT-PCR (qPCR)

Scramble and LUCAT1 siRNA-transfected cells were lysed in TRIzol reagent (Thermo Fisher Scientific, Waltham, MA, USA), RNA was precipitated using the chloroform–isopropanol method and cDNA was synthesized using an iScript cDNA synthesis kit (Bio-Rad, Hercules, CA, USA) followed by reverse-transcription polymerase chain reaction (RT-PCR). PowerTrack SYBR Green Master Mix (Thermo Fisher Scientific, Waltham, MA, USA) was used for qPCR as per an earlier protocol. The following primers in Table 1 were used for the RT-PCR and qPCR.

### 2.8. Identification of Side Population Cells

Side population cells were identified using flow cytometry after staining with Hoechst 33342 (Sigma-Aldrich, St Louis, MO, USA). Scramble-control and LUCAT1 siRNA-transfected TNBC cells were stained with 5 µg/mL Hoechst 33342 dye for 90 min. Cell samples were acquired using a BD FACSCelesta™ Cell Analyzer and analyzed using FCS Express version 7.22.0031 (De Novo software, Pasadena, CA, USA).

### 2.9. Immunoblotting

HCC1806 cells were transfected with scramble-control and LUCAT1 siRNA, and whole-cell lysates were prepared using a modified RIPA lysis buffer. An equal amount of protein was resolved on sodium–dodecyl sulfate polyacrylamide gel, transferred onto PVDF membrane, and immunoblotted using specific antibodies.

### 2.10. ALDEFLUOR Assay

ALDH assay was performed using an ALDEFLUOR kit (01700, Stem Cell Technologies, Vancouver, BC, Canada) as per our previous protocol [31]. Scramble-control and LUCAT1 siRNA-transfected TNBC cells were resuspended in ALDEFLOUR buffer and incubated with activated ALDEFLOUR reagent for 30 min in dark at 37 °C. Following the incubation period, ALDH enzymatic activity was measured using flow cytometry following the manufacturer’s protocol. Diethylaminobenzaldehyde (ALDH inhibitor) was included as a negative control [31].

### 2.11. Cell Cycle Analysis

To determine the cell cycle status of TNBC cells upon LUCAT1 silencing, we performed cell cycle analysis following a previously published protocol [33]. Scramble-control and LUCAT1 siRNA-transfected TNBC cells were trypsinized, fixed with 70% ethanol, and stained with 50 µg/mL propidium iodide containing 0.05% RNAse-A. Cell samples were acquired using BD FACSCelesta™ Cell Analyzer and analyzed using FCS Express (De Novo software, Pasadena, CA, USA).

### 2.12. Flow Cytometry

The expression profile of CD49f (BioLegend, San Diego, CA, USA) in scramble-control and LUCAT1 siRNA-transfected HCC1806 cells was analyzed by flow cytometry. Cells (1 × 10^6^) were stained with anti-CD49f antibody following the manufacturer’s specific protocol. Labeled cells were acquired by BD FACSCelesta™ Cell Analyzer and analyzed using FCS Express (De Novo software, Pasadena, CA, USA).

### 2.13. Statistical Analysis

Statistical analyses were exploratory. Analyses were conducted using R and GraphPad Prism 10. Data were normalized if necessary. Pairwise comparisons were made using Student’s t-test. Kaplan–Meier survival curves were constructed for survival outcomes and log-rank testing was conducted to compare survival outcomes between groups. Results were considered to be significant if *p*-value < 0.05.

## 3. Results

### 3.1. Identification of Differentially Expressed lncRNAs in Triple-Negative Breast Cancer

**Figure 1:**
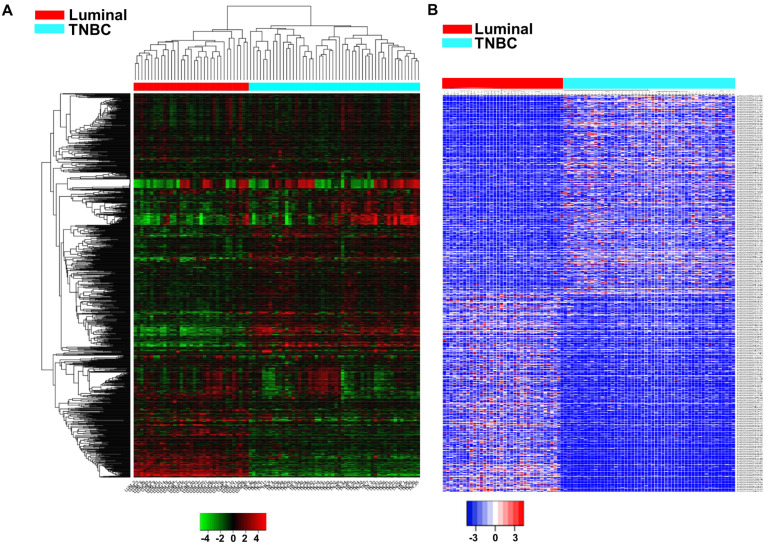
Differentially expressed gene (DEG) profile of protein-coding and noncoding genes. DEG analysis of TNBC vs other subtypes with fold change filter of ≥1.5 and ≤ −1.5 FC was performed. Unsupervised clustering clearly showed two clusters. (**A**) Heat map shows all the variations including protein-coding and noncoding genes between TNBC and all other subtypes (Luminal A, Luminal B, Her2). (**B**) Heat map shows differential expression profile of lncRNA segregated through ENSEMBL-Biomart.

To identify the differentially expressed genes (DEGs) between luminal and TNBC subtypes of breast cancer, we analyzed the GSE192341 dataset comprising 52 TNBC and 35 luminal breast cancer samples. We found a total of 25,680 differentially expressed genes, with 13,576 (53%) upregulated and 12,104 (47%) downregulated in TNBC (Figure 1A, Supplementary Table S1). These DEGs were further screened through ENSEMBL-Biomart https://useast.ensembl.org/info/data/biomart/index.html (accessed on 20 May 2023) to segregate differentially expressed long noncoding RNA (DElncRNA) in TNBC. Our results showed 389 upregulated lncRNAs including novel transcripts, and 386 downregulated lncRNAs including novel transcripts in TNBC (Figure 1B, Supplementary Table S2).

### 3.2. Comprehensive Characterization of LUCAT1 in TNBC and Its Involvement in ceRNA Network

**Figure 2:**
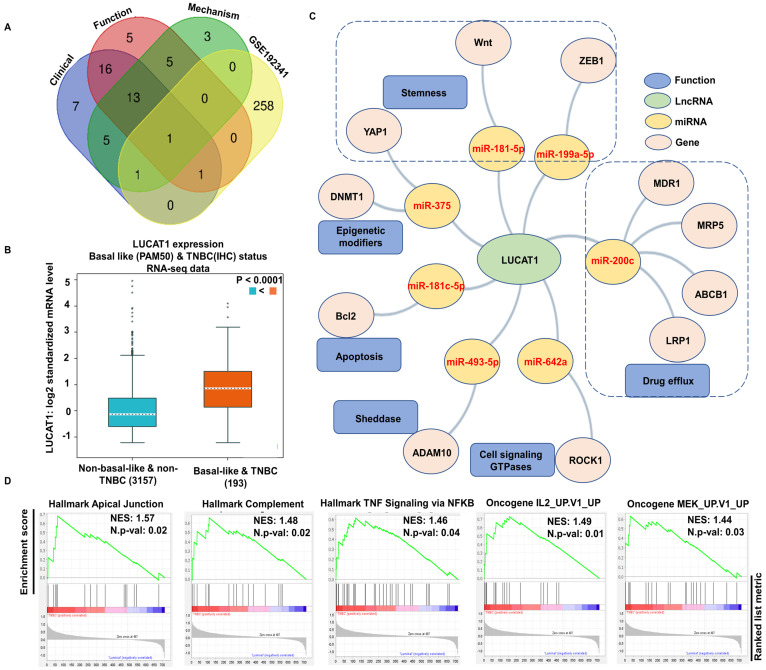
Comprehensive analysis of LUCAT1 in TNBC. (**A**) The list of DElncRNA from GSE192341 was compared to the lnc2cancer3.0 database, which is a collection of experimentally validated mechanistic, functional, and clinically relevant lncRNAs. Venn diagram shows comparative result with GSE192341 and only LUCAT1 was common in all the 3 categories (mechanism, function, and clinical). (**B**) LUCAT1 expression in TNBC and non-TNBC samples. Outliers or extreme values are shown as * in the figure. (**C**) ceRNA network of LUCAT1 using lncACTdb database. (**D**) GSEA of hallmark in cancer (C1; MSigDb) and oncogene signature pathways (C6; MSigDb) with LUCAT1-correlated genes (r^2^ ≥ 0.3) show a significant enrichment in hallmark apical junction pathway (NES:1.57), complement pathway (NES: 1.48), TNF signaling via NFKB (NES: 1.46), oncogene IL2 (NES:1.49), and MEK (1.44) upregulation signatures.

We further evaluated the TNBC DElncRNAs showing a fold change of ≥1.5 (n = 261, excluding novel transcripts) using the lnc2cancer 3.0 database to identify the lncRNAs that might be associated with TNBC at a mechanistic, functional, and clinical level. This database provides experimentally validated information on lncRNAs and circular RNAs (circRNAs) associated with human cancers. Out of 261 differentially expressed lncRNAs in TNBC, only LUCAT1 showed mechanistic, functional, and clinical relevance to TNBC, while lncRNA NRD1 showed clinical and functional relevance to TNBC and LINC01133 had clinical and mechanistic relevance to TNBC (Figure 2A and Supplementary Table S3). As LUCAT1 was comprehensively relevant to all three categories (functional, clinical, and mechanistic), it emerged as the primary lncRNA of interest for further investigation. Next, we analyzed the expression of LUCAT1 using publicly available datasets through bc-GenExMiner, consisting of 193 TNBC samples and 3157 non-TNBC samples. We observed a significantly higher LUCAT1 expression in TNBC samples compared to non-TNBC samples (*p* < 0.0001) (Figure 2B).

The competing endogenous RNA (ceRNA) network is used to evaluate how miRNA and lncRNA affect mRNA’s transcriptional regulation. To evaluate LUCAT1 (lncRNA of our interest) further, we constructed a ceRNA network of LUCAT1 using the lncACTDb database. This allowed us to identify the interactions between LUCAT1 and various miRNAs and mRNAs. We observed LUCAT1 to be associated with the following miRNA-mRNA pairs: miR-375-YAP1-DNMT1, miR-181-5p-WNT, miR-199a-5p-ZEB1, miR-200c-LRP1-ABCB1-MDR1-MRP5, miR-642a-ROCK1, miR-493-5p-ADAM10, and miR-181c-5p-BCL2 (Figure 2C). The results obtained from the ceRNA network suggested that LUCAT1 participates in a highly interconnected ceRNA network of miRNAs and their target mRNAs. Then, we performed a correlation analysis between LUCAT1 and differentially expressed genes in TNBC using the GSE192341 dataset (Supplementary Table S4). Genes with a correlation coefficient (r^2^) greater than 0.3 were selected for further investigation. Gene set enrichment analysis using the MSigDB hallmark gene signature collection (C1) revealed a significant enrichment of HALLMARK_APICAL_JUNCTION, HALLMARK_COMPLEMENT and HALLMARK_TNFA_SIGNALING_VIA_NFKB pathways, with a net enrichment score (NES) of 1.573, 1.48, and 1.46, respectively (Figure 2D and Supplementary Table S5). Similarly, the GSEA using MSigDB oncogene signature (C6) demonstrated a significant enrichment of IL2 and MEK UP gene signature with an NES of 1.49 and 1.44, respectively (Figure 2D). These findings underscore the potential role of LUCAT1 in modulating key cancer-related pathways through its interactions with tumor-suppressive miRs and their target pro-cancer mRNAs, highlighting its relevance in the TNBC progression.

### 3.3. LUCAT1 Induces Growth, Proliferation, and Migration of Triple-Negative Breast Cancer Cells

**Figure 3:**
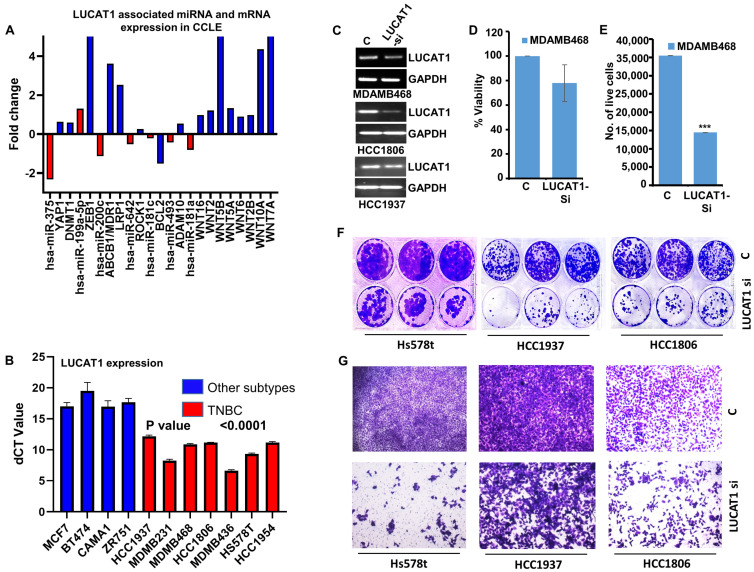
LUCAT1 induces growth, proliferation and migration of TNBC cells. (**A**) Bar graph shows the expression profile of LUCAT1-associated miRNA (red bars) and mRNA (blue bars) in CCLE data of multiple breast cancer cell lines (TNBC vs. all other subtypes). (**B**) Bar graph shows the real-time mRNA expression profile of LUCAT1 in breast cancer subtype-specific cell lines. (**C**) TNBC cells were silenced for LUCAT1. RT-PCR results show mRNA expression level of LUCAT1 and GAPDH in TNBC cell lines. (**D**) MDA-MB-468 cells were silenced for LUCAT1 expression using siLUCAT1. The cell viability was evaluated using MTT assay and the data presented as % viability showing the mean viability ± SD of 6 independent replicates. (**E**) Trypan blue live–dead staining of MDA-MB-468 cells transfected with scramble-control and LUCAT1 siRNA was conducted. The bar graph shows the number of live cells only. *** *p* = 1.65046 × 10^−6^. (**F**) TNBC cells were silenced for LUCAT1 expression. Representative pictures of colony-formation assay in TNBC cells in the presence and absence of LUCAT1 are shown. (**G**) TNBC cells were silenced for LUCAT1 expression and subjected to transwell migration assay. Representative images show the cells migrated through the upper chamber in scramble-control and LUCAT1 siRNA group. Images captured at 10× magnification.

Next, we performed in silico validation of the LUCAT1-regulated ceRNA network using the CCLE dataset to query the expression of suppressive miRs and their associated mRNAs in multiple TNBC cell lines. A total of 21 TNBC and 29 other subtypes of breast cancer cell lines were analyzed for differential gene expression of miRNAs and mRNAs forming a LUCAT1 ceRNA network (Supplementary Table S6). We observed that the decreased expression of LUCAT1-regulated hsa-miR-181a (hsa-miR-181-5p) was associated with an increased expression of WNT family oncogenes (WNT16, WNT2, WNT5B, WNT5A, WNT6, WNT2B, WNT10A and WNT7A). Similarly, a reduced expression of hsa-miR-493-5p and hsa-miR-642 was observed along with an elevated expression of ADAM10 and ROCK1, respectively. In addition, reduced expression of hsa-miR-375 and hsa-miR-200c was noted with an increased expression of YAP1/DNMT1 and MDR1/LRP1, respectively (Figure 3A). Inspired by our in silico analysis indicating the involvement of LUCAT1 in TNBC, we assessed the role of LUCAT1 in TNBC cells in vitro. The baseline expression analysis of LUCAT1 in multiple breast cancer cells showed a higher expression of LUCAT1 in TNBC cells compared to other subtypes (Figure 3B). To further confirm the functional importance of LUCAT1 in TNBC, its impact on growth, proliferation, and migration was evaluated. Through the virtue of modulating the anti-cancer miRNA-oncogenic mRNA, LUCAT1 can act as a stimulator of cancer growth. Indeed, inhibition of LUCAT1 in several TNBC cell lines (Figure 3C) showed that LUCAT1 silencing significantly decreased the viability of MDA-MB-468 cells (Figure 3D). The trypan blue dye exclusion assay revealed greater than twofold reduction in MDA-MB-468 viable cells with LUCAT1 silencing (Figure 3E). Next, we examined the impact of LUCAT1 silencing on the clonogenic potential of TNBC cells and found that LUCAT1 silencing significantly reduced the colony-forming ability of Hs578t, HCC1937, and HCC1806 cells (Figure 3F). Some of the target genes in the LUCAT1 ceRNA network have been known to associate with the elevated migratory potential of cancer cells, and hence we queried the impact of LUCAT1 silencing on the migratory potential of TNBC cells. LUCAT1 silencing indeed reduced the transwell migration of TNBC cells compared to the control group (Figure 3G). These data indicate the involvement of LUCAT1 in growth, proliferation, and migration of TNBC cells.

### 3.4. LUCAT1 Functions as a Central Node Regulating Drug Efflux, Stemness, and Programmed Cell Death in Triple-Negative Breast Cancer Cells

**Figure 4:**
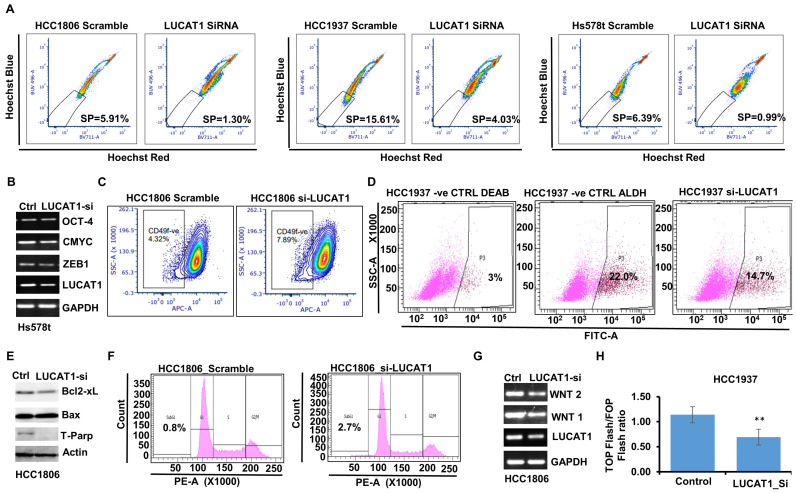
LUCAT1 functions as an important axis regulating drug efflux, stemness, and programmed cell death in TNBC cells. (**A**) TNBC cells were silenced for LUCAT1. Identification of side population (SP) cells from multiple TNBC cells. SP = side population. SP cells of HCC1806, HCC1937 and Hs578t were analyzed by dual-wavelength flow cytometer after staining with Hoechst 33342. (**B**) Hs578t cells were silenced for LUCAT1, and mRNA-expression level of stemness markers (OCT-4, CMYC, and ZEB1) were examined. (**C**) HCC1806 cells were silenced for LUCAT1, and CD49F level was examined using FACS analysis. (**D**) HCC1937 cells were silenced for LUCAT1 and ALDH activity assay was performed. (**E**) Total protein lysates from HCC1806 cells transfected with scramble-control and LUCAT1-siRNA were immunoblotted for the expression of BAX, Bcl2-xL, and T-PARP. (**F**) Flow cytometry analysis of cell distribution in respective phases of cell cycle of HCC1806 cells transfected with scramble-control and LUCAT1 siRNA. Sub-G1 cells correspond to apoptotic cells. (**G**) mRNA expression level of WNT1, WNT2, LUCAT1 and GAPDH in HCC1806 cells transfected with scramble-control and LUCAT1 siRNA. (**H**) TOPflash/FOPflash reporter assay in HCC1937 cells transfected with scramble-control and LUCAT1 siRNA (** *p* = 0.00252).

**Figure 5:**
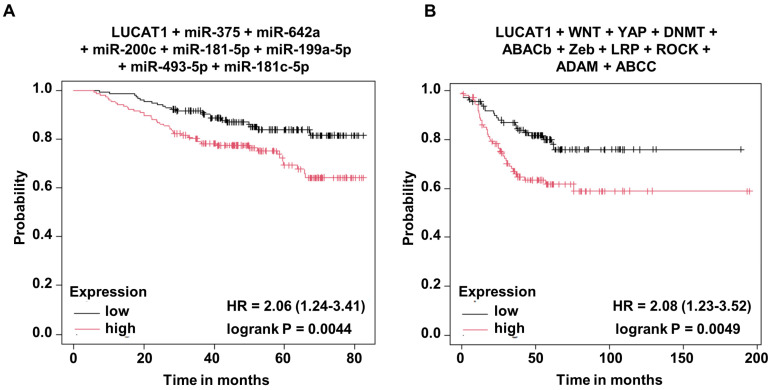
Association between LUCAT1 and miRNA, and LUCAT1 and mRNAs in TNBC. (**A**) Kaplan–Meier plots of triple-negative breast cancer survival in relation to high LUCAT1 + low miRs (miR-375 + miR-642a + miR-200c + miR-181-5p + miR-199a-5p + miR-493-5p + miR-181c-5p). (**B**) Kaplan–Meier plots of triple-negative breast cancer survival in relation to high LUCAT1 + high target genes (WNT + YAP + DNMT + ABACb + Zeb + LRP + ROCK + ADAM + ABCC).

Since *in-silico* investigations suggested the involvement of LUCAT1 in regulating genes related to drug efflux, stemness, and programmed cell death in TNBC cells (Figure 2C), we initiated a comprehensive set of experiments to validate the *in silico* observations. To evaluate the role of LUCAT1 in enhancing the drug efflux efficacy of TNBC cells, we performed a side population assay using Hoechst 33342 dye. Our results indicated that silencing of LUCAT1 significantly reduced the side population cells in TNBC cells. Approximately fourfold reduction in the side population cells was noted in LUCAT1-silenced HCC1806 and HCC1937 compared to respective scramble-control cells (4.03% in LUCAT1_si HCC1937 versus 15.61% in scramble-control HCC1937; 1.30% in LUCAT1_si HCC1806 versus 5.91% in scramble-HCC1806). Similarly, greater than sixfold reduction in the side population cells was observed upon LUCAT1 silencing in Hs578t cells compared to scramble-control cells (Figure 4A). Next, we wanted to evaluate the stemness potential of TNBC cells in the presence and absence of LUCAT1. We observed that a decrease in LUCAT1 expression reduced the level of stemness-associated factors- OCT4, cMYC, and ZEB1 in Hs578t cells compared to control cells (Figure 4B). Decreased expression of stemness-associated genes upon LUCAT1 modulation prompted us to evaluate the impact of LUCAT1 silencing on the expression level of the stemness marker CD49f and identify the population of cancer stem cells/progenitor cells in TNBC. An approximate twofold increase in the CD49f-negative population was noted in the absence of LUCAT1 (7.89%) compared to scramble-control (4.32%) HCC1806 cells (Figure 4C). The results from the ALDEFLUOR assay supported our hypothesis that LUCAT1 contributes to TNBC stemness. We found an approximate 1.5-fold reduction in the progenitor cell population upon LUCAT1 silencing compared to scramble-control in HCC1937 cells (22.0% in control versus 14.7% in LUCAT1-siRNA-treated cells) (Figure 4D). Next, we evaluated the effect of LUCAT1 silencing on key protein markers of programmed cell death in TNBC. Immunoblotting of total protein lysates from cells treated with control or siLUCAT1 showed a decreased level of anti-apoptotic Bcl2-xL and total PARP with a minor increase in pro-apoptotic Bax expression (Figure 4E). Cell cycle analysis of HCC1806 cells exhibited increased sub-G1 population upon silencing of LUCAT1 (0.8% in scramble-control compared to 2.7% in LUCAT1 siRNA treated HCC1806 cells) (Figure 4F). Our in silico analysis also indicated the contributory role of LUCAT1 in inducing WNT cascade via sponging miR-181a-5p. The WNT signaling pathway confers multi-drug resistance, enhances DNA damage repair, maintains cancer progenitor population, and induces growth and proliferation in cancer cells [34]. In order to determine the involvement of LUCAT1 in inducing WNT signaling in TNBC cells, we examined the expression level of WNT1 and WNT2 in LUCAT1-silenced HCC1806 cells. Our data revealed a decrease in the expression level of both WNT1/2 upon LUCAT1 silencing compared to scramble-control HCC1806 cells (Figure 4G). To evaluate whether LUCAT1 induces the WNT/ẞ-catenin-regulated transcription factor TCF/LEF, a luciferase-based reporter assay was carried out to measure the TCF/LEF transcription factor activity using TOP Flash/FOP Flash constructs. LUCAT1 silencing decreased the relative luciferase activity (TOP Flash/FOP Flash) of HCC1937 cells, confirming the contributory role of LUCAT1 in inducing the WNT signaling pathway (Figure 4H). Next, we interrogated the association of LUCAT1 and its ceRNA network composed of miRs and mRNAs with overall survival of triple-negative breast cancer patients. High expression of LUCAT1 and low expression of miR-375, miR-642a, miR-200c, miR-181a-5p, miR-199a-5p, miR-493-5p, and miR-181c-5p were associated with poor overall survival in TNBC patients (Figure 5A). We also noted that high expression of LUCAT1 and high expression of ceRNA network target genes (WNT + YAP + DNMT + ABACb + Zeb + LRP + ROCK + ADAM + ABCC) exhibited poor survival in TNBC patients (Figure 5B). Together, these data support the role of LUCAT1 in modulating the side population, stemness-associated features, apoptosis-related features and the WNT pathway in TNBC cells and provide supportive functional evidence for the LUCAT1 ceRNA network in TNBC.

## 4. Discussion

Owing to the aggressive progression of TNBC, there has been sustained interest in examining the potential nodes that may help with prognostics and/or treatment of TNBC. In this line, expression patterns of noncoding RNAs (including miRNAs, lncRNAs, and circRNAs) have been assessed along with their putative targets, and their functional importance have also been tested in TNBC. LncRNAs, especially, can exert various functions in the nucleus as well as cytoplasm. Nuclear-localized lncRNAs regulate gene expression via epigenetic modification [35,36,37], DNA methylation [38], chromatin remodeling [39,40], and by interacting with nuclear proteins [41] and transcription factors [42,43]. However, cytoplasmic lncRNAs regulate gene expression post-transcriptionally and post-translationally by interacting with cytoplasmic proteins [44] and regulating mRNA metabolism by interacting with miRNAs [45,46]. As lncRNAs regulate gene expression, both at nuclear and cytoplasmic level, their contribution in normal physiology as well disease state including cancer cannot be denied. In fact, lncRNAs participate in various aspects of cancer initiation and progression, including cancer cell proliferation, invasion, and migration, as well as development of drug resistance. The current study emphasizes the involvement of lung cancer-associated transcript 1 (LUCAT1), an lncRNA, in TNBC. We identified 25,680 DEGs in TNBC, with a considerable portion being lncRNAs. Among these, 389 lncRNAs were found to be upregulated and 386 downregulated in TNBC. LUCAT1, an oncogenic lncRNA, emerged as the lncRNA of interest due to its comprehensive relevance across clinical, mechanistic, and functional aspects of TNBC. Analysis of publicly available datasets confirmed the significant overexpression of LUCAT1 in TNBC samples compared to non-TNBC samples, highlighting its potential importance in TNBC. This observation was also corroborated with TNBC cell lines. These results are in line with earlier findings where elevated LUCAT1 expression was observed in breast cancer [47] and LUCAT1 upregulation in TNBC correlated with poor prognosis. LUCAT1 negatively impacts miR-5702 and induces proliferation of TNBC cells [48]. Another study showed that LUCAT1 contributes to a positive feedback loop impacting ELAV-like RNA-binding protein 1 (ELAVL1), lin-28 homologue B (LIN28B) and SRY-box transcription factor 2 (SOX2) [49]. In fact, LUCAT1 has been found to be upregulated in multiple cancers, including non-small-cell lung carcinoma (NSCLC), esophageal cancer, renal cell carcinoma, and osteosarcoma [48,50,51,52,53,54]. In NSCLC, LUCAT1 associates with polycomb repressor complex (PRC2) and regulates the cell cycle [50]. Silencing of tumor suppressors via methylation contributes to carcinogenesis, and LUCAT1 can impact this process by virtue of regulating DNA methyl transferase 1 (DNMT1) [51]. LUCAT1 also impacts several signal transduction pathways in multiple cancer types [48,50,51,52,53,54]. These studies highlight the importance of LUCAT1 in carcinogenesis. 

Adding functional importance to the lncRNAs, the competing endogenous RNA (ceRNA) hypothesis, which suggests that lncRNAs may carry one or more miRNA response elements (MREs), effectively inhibit miRNA function and communicate with mRNAs [15], has acquired increased interest. Though some recent studies have examined the ceRNA networks in TNBC [55,56,57], no studies have identified a LUCAT1-ceRNA network in TNBC. Hence, we evaluated the LUCAT1-mediated ceRNA network in TNBC and observed intricate interactions with multiple miRNAs and mRNAs. The association of LUCAT1 with miRNAs such as miR-375, miR-642a, miR-200c, miR-181a-5p, miR-199a-5p, miR-493-5p, and miR-181c-5p, which in turn regulate key oncogenes (e.g., YAP1, DNMT1, WNT family members, ZEB1, MDR1, MRP5, ABCB1, LRP1, ROCK1, ADAM10 and BCL2), suggests that LUCAT1 may play an important role in TNBC. ceRNA network of LUCAT1 suggests its contribution in key events like stemness, drug efflux, GTPase mediated cell signaling, programmed cell death, and epigenetic modifications, supporting the oncogenic role of LUCAT1 in TNBC. Gene set enrichment analysis (GSEA) further indicated the involvement of LUCAT1 in cancer hallmark pathways, such as APICAL_JUNCTION, COMPLEMENT, and TNFA_SIGNALING_VIA_NFKB. These pathways are known to be essential for tumorigenesis, metastasis [58], and immune response modulation [59], highlighting the potential influence of LUCAT1 in TNBC. In fact, higher expression of genes associated with apical junction pathway leads to poor outcome and increased metastasis, angiogenesis and proliferation of cancer cells [58]. Complement system includes inflammatory response and adaptive immune response which have a proven role in tumorigenesis [60] but due to limited information, we did not find any known direct association of LUCAT1 with complement pathway. While LUCAT1 regulates NF-KB signaling in chronic obstructive pulmonary disease (COPD) or inflammatory bowel disease (IBD), its specific involvement in cancer-related NF-KB signaling remains unknown [59]. 

Further, we analyzed the CCLE dataset for *in silico* validation of LUCAT1-ceRNA network components, and observed a correlation between decreased expression of LUCAT1-sponged miRNAs (miR-375, miR-200c, miR-642, miR-493-5p, and miR-181a) and increased expression of their target oncogenes (YAP1, DNMT1, MDR1, LRP1, ROCK1, ADAM10 and WNT family genes). The validation data encouraged us to conduct *in vitro* functional assays confirming the oncogenic potential of LUCAT1 on TNBC cells. We observed that LUCAT1 silencing resulted in significant reductions in cell viability, colony-forming ability, and migration. These findings align with the observed decrease in stemness markers (e.g., OCT4, CMYC, ZEB1, CD49f) and progenitor cell populations upon LUCAT1 silencing. Of note, LUCAT1 performs similar functions in other cancer types as LUCAT1 silencing significantly inhibits papillary thyroid cancer cell proliferation and invasion as well as induces apoptosis [61]. Significant upregulation of LUCAT1 in clear cell renal cell carcinoma (ccRCC) samples has been reported, and in fact, LUCAT1 overexpression induces proliferation, migration and invasion of ccRCC cells by inducing AKT and suppressing GSK3β. LUCAT1-miR connections are important as the LUCAT1 and miR-181a-5p interaction using reporter assay showed that overexpression of miR-181a-5p inhibits LUCAT1-induced cell proliferation [47]. Similarly, LUCAT1 increases TNBC cell growth, migration, invasion and EMT by negatively regulating miR-5702 [48]. Significant upregulation of LUCAT1 induces proliferation, migration and invasion of breast cancer cells acting as a molecular sponge for miR-7-5p [54]. LUCAT1 also seems to contribute to drug efflux pathways that may impact chemoresistance, a major challenge in TNBC treatment. WNT signaling pathway contributes to chemoresistance [62]; induces DNA damage repair pathways [63], and maintain cancer stem cells. Decreased expression of WNT1/2 and reduced TCF/LEF transcription factor activity upon LUCAT1 silencing emphasizes the contributory role of LUCAT1 in WNT pathway activation in TNBC cells. 

## 5. Conclusions

The results presented here demonstrate the presence of a ceRNA network involving lncRNA LUCAT1 and various miRs along with their gene targets with oncogenic functions in TNBCs in comparison to luminal subtypes. LUCAT1-ceRNA network may have functional importance as LUCAT1 inhibition leads to reduction in growth, migration and stem-like features. Also, high LUCAT1 and low associated miR(s) as well as high LUCAT1 and high associated mRNA(s) expression levels associated with poor overall survival in TNBC patients. In summary, we propose that LUCAT1 via its ceRNA network may have prognostic significance and impact various biological functions that contribute to TNBC growth and progression.

## Figures and Tables

**Table 1:** Primer sequences for PCR and qPCR.

Primer	Forward 5′-3′	Reverse 5′-3′
LUCAT1	GCTCGGATTGCCTTAGACAG	GGGTGAGCTTCTTGTGAGGA
GAPDH	AATCCCATCACCATCTTCCA	TGGACTCCACGACGTACTCA
Actin1	ACCATGGATGATGATATCGC	ACATGGCTGGGGTGTTGAAG
OCT-4	GTTGATCCTCGGACCTGGCTA	GGTTGCCTCTCACTCGGTTCT
cMYC	TCAAGAGGCGAACACACAAC	GGCCTTTTCATTGTTTTCCA
Wnt1	GGGTCCTCCTAAGTCCCTTC	CCAACCTCATTTCCACATCAT
Wnt2	CGGGAATCTGCCTTTGTTTA	TTCCTTTCCTTTGCATCCAC
ZEB1	CCTGAAATCCTTAATCCTCCGC	TGGTTCCTGTTCCTAGTGGG

## Data Availability

The original contributions presented in this study are included in the article/Supplementary material. Further inquiries can be directed to the corresponding author.

## Supplementary Materials

The following supporting information can be downloaded at: https://www.mdpi.com/article/10.3390/cells13221918/s1, Data files are added as Supplementary Tables S1–S6. Supplementary Table S1: DEG_GSE192341. Supplementary Table S2: DEG_lncRNA. Supplementary Table S3: predicted_miRNA-lncRNA targets. Supplementary Table S4: TCGA_miRNA DEG. Supplementary Table S5: GSE96058_DESeq2 results. Supplementary Table S6: CCLE_mRNA_DE_miRNA.
